# Comment on Panuccio et al. Quality of Assessment Tools for Aphasia: A Systematic Review. *Brain Sci.* 2025, *15*, 271

**DOI:** 10.3390/brainsci15111233

**Published:** 2025-11-17

**Authors:** Sarah J. Wallace, Katerina Hilari, Klaus Willmes, Marcus Meinzer, Claudia Peñaloza, Lizet van Ewijk, Rebecca Palmer, Sally Zingelman, William D. Hula, Caterina Breitenstein

**Affiliations:** 1 Queensland Aphasia Research Centre, The University of Queensland, Brisbane 4029, Australia; s.zingelman@uq.edu.au; 2 Surgical Treatment and Rehabilitation Service (STARS) Education and Research Alliance, The University of Queensland and Metro North, Brisbane 4029, Australia; 3 School of Health and Medical Sciences, Centre for Language and Communication Science Research, City St George’s, University of London, London EC1V 0HB, UK; k.hilari@citystgeorges.ac.uk; 4 Department of Neurology, RWTH Aachen University Hospital, 52074 Aachen, Germany; willmes@neuropsych.rwth-aachen.de; 5 Department of Neurology, University Medicine Greifswald, 17489 Greifswald, Germany; marcus.meinzer@med.uni-greifswald.de; 6 Department of Cognition, Development and Educational Psychology, Institute of Neurosciences, University of Barcelona, 08035 Barcelona, Spain; claudia_penaloza@ub.edu; 7 Research Group Speech and Language Therapy, Research Center Healthy and Sustainable Living, HU University of Applied Sciences, 3584CS Utrecht, The Netherlands; lizet.vanewijk@hu.nl; 8 Sheffield Centre for Health and Related Research, University of Sheffield, Sheffield S10 2TN, UK; r.l.palmer@sheffield.ac.uk; 9 Geriatric Research, Education, and Clinical Center, VA Pittsburgh Healthcare System, Pittsburgh, PA 15240, USA; william.hula@va.gov; 10 Audiology and Speech Pathology Service, VA Pittsburgh Healthcare System, Pittsburgh, PA 15240, USA; 11 Department of Communication Science and Disorders, University of Pittsburgh, Pittsburgh, PA 15260, USA; 12 Department of Neurology, University of Muenster, 48149 Muenster, Germany; breitens@uni-muenster.de

We write on behalf of the Collaboration of Aphasia Trialists and in response to an article recently published in the journal *Brain Sciences*: Quality of Assessment Tools for Aphasia: A Systematic Review by Panuccio and colleagues [1]. While we applaud the authors’ efforts to provide a comprehensive review of aphasia measurement instruments, we have identified numerous significant methodological concerns and factual errors that undermine the quality, validity, and utility of this review. We have outlined some of these concerns below. In the interest of providing a timely response, these constitute only selected examples.

First, an outdated version of the COSMIN quality rating criteria [2] is used instead of the more recent checklist [3], and there are substantial errors in its application, including that, in many cases, citations do not support the ratings made. For example:

- The study evaluating the Turkish version of the Aphasia Rapid Test (ART) [4] is awarded the highest quality rating of all measurement instruments in the systematic review, with uniformly positive ratings across 9 of 10 COSMIN criteria, despite the supporting paper only evaluating one aspect of one of the COSMIN quality criteria (inter-rater agreement as one aspect of reliability).
- The paper reporting on the adaptation of the Stroke Specific Quality of Life scale (SS-QOL, Williams et al., 1999) [5] to develop an aphasia-adapted version, the English language Stroke and Aphasia Quality of Life Scale (SAQOL-39) and test its content validity [6] is listed as a Dutch publication in Table 2 and not considered in Table 3 for the development of the SAQOL-39. The structural validity of the original English-language SAQOL-39 [7] and SAQOL-39g [8] is rated as insufficient despite both studies reporting results of Exploratory Factor Analysis, while the Japanese SAQOL-39 [9] received a positive rating despite no reported factor analysis at all in the cited article.
- The Aphasia Communication Outcome Measure (ACOM) was rated negatively for internal consistency, even though the cited paper [10] reports an IRT- based marginal reliability coefficient, an internal consistency measure.
- For psychometric evaluation of the original German-language Communicative Activity Log (CAL), the authors refer to an evaluation study for the Korean version of the CAL [[11], Table 2], which does not include any data for the German CAL, and merely cites a review article for the German CAL standardization. This review article includes the CAL questions in an appendix, without reporting any psychometric data.
- Ratings for the Communication Participation Item Bank (CPIB) [12] do not accurately reflect available information on Patient Reported Outcome Measure (PROM) development or psychometric information related to item response theory (IRT) analyses [12].
- In many cited articles that include general stroke samples, the proportion of people with aphasia is not specified, for example, for the German-language screening (LAST) [13]. It therefore remains unclear whether the corresponding measurement instrument has even been evaluated in the target population (people with aphasia) at all.

These examples raise significant questions about the rigour of the quality assessment process and erode the confidence we can have in the findings. Furthermore, Table 3 lacks documentation supporting the authors’ quality ratings, making it impossible to verify their judgments.

Second, the review is limited by selection bias, having excluded published test manuals containing robust standardization data for well-established aphasia measures. Previous reviews on aphasia assessment instruments have underscored that search strategies restricted to just research databases (i.e., peer-reviewed articles) are likely to miss available psychometric data [14,15,16]. Similarly, other systematic review authors have noted that “comprehensive language assessments often report their psychometric properties within their purchased test manuals or through online sources and not within peer-reviewed journals” [[17], p. 3] leading them to refine their search strategy to include test manuals and other sources to access their psychometric data. In this view, the review by Panuccio and colleagues [1] shows notable omissions, including the test manuals for:

- The German-language Aachen Aphasia Test (AAT) [18] and Scenario Test [19], and the Dutch Amsterdam-Nijmegen Everyday Language Test (ANELT) [20] all of which contain substantial psychometric data, with the AAT being one of the most extensively psychometrically evaluated measures in the field.
- The original English version of the Comprehensive Aphasia Test (CAT) [21], a significant oversight given the CAT’s increasing importance in aphasia research over the past two decades and the multiple adaptations it has inspired [22].

Third, the structure used to categorize measurement instruments (Table 2) lacks coherence, sometimes referring to constructs, sometimes to target populations, and other times to combinations of populations and recovery phases. The fundamental distinction between language and communication is not recognized in this structure, a significant oversight when it comes to aphasia measurement instruments. Accordingly, measures of communication, e.g., the Scenario Test [19], Aphasia Communication Outcome Measure ACOM (Hula et al., 2015) [10], and American Speech-Language and Hearing Association Functional Assessment of Communication Skills for Adults [23], are miscategorized as measures of language. Within the category structure, there are numerous further examples of measurement instruments being incorrectly categorized. Such examples included the following:

- The Apraxia of Speech Rating Scale [24] is categorized as a language measure when it assesses apraxia of speech, a *motor speech disorder*.
- The Abbey Pain Scale [25] is categorized as a measure of language, when it measures *pain*.
- The Auditory-Perceptual Rating of Connected Speech in Aphasia [26] is categorized as an “Auditory-perceptive” measure, rather than a multidimensional measure of *connected speech performance*.
- The ACOM [10] is categorized as a quality-of-life measure, when its authors specifically identify it as a patient-reported measure of *communicative function*.
- The CPIB [12] is categorized as a quality-of-life scale, rather than a measure of *communicative experience.*

Finally, throughout the paper, there are numerous referencing errors, e.g., Kavakci et al., (2022) [4], which is reference #65 in the reference list in Panuccio et al. [1], is cited as reference #66 in the text, or measures are attributed to the wrong author team, e.g., the SS-QOL [developed by Williams et al. (1999) [5]] is attributed to Hilari (2001) ref #299 in Table 2 and to Northcott (2013) ref 298 in Table 3. These errors impede the reader’s ability to link ratings with supporting evidence, making verification difficult and reducing confidence in this review’s ratings.

The issues in Panuccio et al. (2025) [1], at best, undermine the review’s utility as a guide to measurement instrument selection, and, at worst, provide information which could compromise future aphasia research design and clinical outcomes. Low-quality systematic reviews of assessment and outcome measurement instruments can have many negative consequences, including the following:

1. **Producing misleading conclusions** about the psychometric quality of measurement instruments, which may misinform decision-making in healthcare and research.
2. **Hindering the development of effective interventions or treatments** if unreliable and invalid measurement instruments are selected as outcome measures.
3. **Negatively affecting patient care** by impacting aphasia assessment guidelines, which could lead to incorrect diagnoses, poor treatment choices, and worse health outcomes.

Over more than ten years, the Collaboration of Aphasia Trialists’ members (>300 across >40 countries) have made painstaking efforts to improve the quality of aphasia research. These include multiple initiatives to improve the quality, efficiency, and global relevance of measurement instruments and practices [22,27,28,29,30,31,32,33,34,35,36]. As a collaboration focused on enhancing the quality and reporting of aphasia research, we are compelled to draw attention to the issues in this paper. The authors’ endeavour to critically evaluate the quality of available aphasia tests within the framework of a systematic review is highly commendable. However, given the potential impacts outlined above, we recommend that the authors review and revise the manuscript.
